# P221: The value of administrative data for the surveillance of healthcare-associated infections: a systematic review

**DOI:** 10.1186/2047-2994-2-S1-P221

**Published:** 2013-06-20

**Authors:** MS Van Mourik, PJ van Duijn, KG Moons, MJ Bonten

**Affiliations:** 1 Department of Medical Microbiology, University Medical Center Utrecht, Utrecht, The Netherlands; 2 Julius Center for Health Sciences and Primary Care, University Medical Center Utrecht, Utrecht, The Netherlands

## Introduction

Administrative data are widely used in the surveillance of medical outcomes including healthcare-associated infections (HAI). The validity of administrative data-based surveillance, however, has been questioned.

## Objectives

This systematic review assesses the validity of using administrative data for the surveillance of HAI compared to traditional methods.

## Methods

A comprehensive literature search (Jan 1995 – March 2012) was conducted in Medline, Embase and CINAHL. All retrieved titles were screened for relevance and selected studies underwent quality assessment (using QUADAS-2) and data-extraction by two reviewers. Studies were stratified by HAI type and methodological robustness. Where possible, bivariate modeling was used to generate summary estimates of sensitivity and specificity and predictive values were assessed. Subgroup analyses were performed to identify determinants of performance.

## Results

The search yielded 8474 unique titles, 10 articles were published after the search date and 4 were identified through cross-referencing. Ultimately, 57 studies were retained for analysis. Methodology used was highly variable and 29 of 57 studies suffered from (at least some) partial or differential verification bias; furthermore, only 32 studies provided sufficient data for assessment of all performance measures. In these studies, the pooled sensitivity ranged from 18.4% to 62.7% depending on the type of HAI targeted and specificity ranged from 91.5% to 98.8%. Positive predictive value was highly variable (0 to 96%). Performance varied significantly by study quality and HAI type.

## Conclusion

Surveillance of HAI using administrative data is in poor agreement with traditional surveillance methods, both in terms of sensitivity and positive predictive value. Furthermore, the study design of studies investigating the value of administrative data surveillance is sometimes suboptimal and results should be interpreted with care.

## Disclosure of interest

None declared

